# Is it all about the feeling? Affective and (meta-)cognitive mechanisms underlying the truth effect

**DOI:** 10.1007/s00426-020-01459-1

**Published:** 2021-01-23

**Authors:** Annika Stump, Jan Rummel, Andreas Voss

**Affiliations:** Institute of Psychology, Heidelberg University, Hauptstrasse 47-51, 69117 Heidelberg, Germany

## Abstract

People are more likely to judge repeatedly perceived statements as true. A decisive explanation for this so-called truth effect is that the repeated information can be processed more fluently than new information and that this fluency experience renders the information more familiar and trustworthy. Little is known, however, regarding whether and how affective states and dispositional cognitive preferences influence the truth effect. To this end, we conducted two experiments in which we manipulated (a) processing fluency via repetition, (b) the time interval (10 min vs. 1 week) between repetitions, and (c) short-term affective states using the presentation of emotional faces (Experiment 1) or the presence of an irrelevant source for changes in affective states (Experiment 2). Additionally, we assessed the dispositional variables need for cognitive closure (NCC), preference for deliberation (PD) and preference for intuition (PI). Results of Experiment 1 showed that the truth effect was significantly reduced for statements that were followed by a negative prime, although this was the case only for the longer repetition lag. Furthermore, higher NCC and lower PD scores were associated with an increased truth effect. Results of Experiment 2 replicated the moderating role of NCC and further showed that participants, who were provided with an alternative source for changes in their affective states, showed a reduced truth effect. Together, the findings suggest that (a) fluency-related changes in affective states may be (co-)responsible for the truth effect, (b) the truth effect is decreased when the repetition interval is long rather than short, and (c) the truth effect is increased for individuals with a higher need for cognitive closure. Theoretical implications of these findings are discussed.

## Introduction

We receive large amounts of information every day. Digitalization and the growing use of new channels, such as social networks, are steadily increasing the number of daily information. However, as currently publically discussed under the catch phrase “fake news”, not all provided information is true. Therefore, we need to evaluate the quality of information, that is, we need to decide, whether the perceived information is trustworthy. But how do we decide this? It has been argued that people often deploy simple heuristics when they make judgments under uncertainty (e.g., Tversky & Kahneman, 1974), and this may also apply to truth judgments. Indeed, it has been shown that familiarity with given information is used as a cue for judging its truthfulness: Familiar information is more likely to be considered as true as compared to unfamiliar information—a phenomenon referred to as truth effect (Dechêne, Stahl, Hansen, & Wänke, 2010; Hasher, Goldstein, & Toppino, 1977).

Since its first demonstration in 1977, the truth effect has been replicated more than 50 times (Dechêne et al., 2010). Given that the truth effect is such a stable phenomenon, a better understanding of this effect seems crucial. Accordingly, the primary aim of the present research was to investigate potential cognitive and affective underpinnings of the truth effect as well as to investigate how dispositional variables, that is, the need for cognitive closure and the preference for making decisions deliberately or intuitively, influence its size. An additional aim was to investigate how these factors are related to metacognitive judgments, more precisely, the judged subjective confidence with which truth judgments are made. Fluency can influence confidence (e.g., Alter, Oppenheimer, Epley, & Eyre, 2007); therefore, it is plausible that repetition (a source of conceptual and perceptual processing fluency) may influence both truth judgments and the subjective confidence with which these truth judgments are made. At present, there are barely any studies that have investigated these effects simultaneously (for an exception, see Koch & Forgas, 2012). However, we assume that perceived fluency does not only cause participants to believe in the truthfulness of a statement but also increases confidence in this judgment. This confidence could then prevent people from searching for additional information and questioning their original judgment. This has obvious practical implications, for example, in the context of fake news.^1^

Most researchers assume that the truth effect is based on the experienced high processing fluency or feeling of familiarity caused by repetition. Of course, processing fluency and familiarity are not identical, but they are closely related concepts: Whereas fluency describes the feeling a person senses when the processing of a stimulus subjectively requires little effort, familiarity can result from a cognitive interpretation of this feeling (Unkelbach, 2007). Studies have further shown that the truth effect can be induced by both the manipulation of conceptual fluency (see e.g., Arkes, Boehm, & Xu, 1991; Hawkins, Hoch, & Meyers-Levy, 2001) and perceptual fluency (see e.g., Reber & Schwarz, 1999; Garcia-Marques, Silva, & Mello, 2016). Hence, a broader definition for the truth effect is an increase of perceived truthfulness for fluently processed information (e.g., Koch & Forgas, 2012). Indeed, the relative processing fluency seems to be decisive for the occurrence of a truth effect: Dechêne, Stahl, Hansen, and Wänke (2009) showed that the truth effect only occurs when fluent and disfluent information is presented intermixed. Fluency is regularly manipulated through the presentation of new and repeated information (e.g., information already presented during a previous phase of the experiment). As Garcia-Marques et al. (2016) have stated, due to the semantic and verbatim repetition, this manipulation can be considered as a source of both conceptual and perceptual processing fluency (see also Garcia-Marques, Silva, Reber, & Unkelbach, 2015).

Whereas it seems clear that fluency provokes a truth effect, the cognitive and affective processes underlying a fluency experience and the resulting truth effect are not well understood. It has been argued that also memory-based processes, such as recognition and source memory, influence judgments of truth (e.g., Law, 1998). However, as Nadarevic and Erdfelder (2014) point out, memory-based explanations rather extend the fluency account and thus do not challenge the core assumption that perceived processing fluency is the central mechanism underlying the truth effect. Unkelbach and Rom (2017) recently proposed a theory aiming to explain the repetition-based truth effect in greater depth. Specifically, they argue that corresponding references in memory assign meaning to a given statement, and a high number of corresponding references, which are linked coherently, result in the fluent processing of a statement. This account provides a comprehensive explanation for the occurrence of a fluency experience. However, not only fluency but also affective processes can trigger a feeling of familiarity and influence truth judgments. Garcia-Marques, Mackie, Claypool, and Garcia-Marques (2004), for example, found that perceived positivity alone signaled familiarity (Experiment 2) and further that a positive mood state led to more “true” judgments (positive vs. neutral mood state, Experiment 3). Claypool, Hall, Mackie and Garcia-Marques (2008) showed that a (mis)attribution process is crucial to the positivity-cues-familiarity effect. In their study, participants who could—after a positive mood induction—attribute their current mood state to an external source showed no positivity-cues-familiarity effect.

Positive affect has not only been linked to familiarity, but also to high processing fluency (see e.g., Winkielman & Cacioppo, 2001; Winkielman, Schwarz, Fazendeiro, & Reber, 2003; Topolinski, Likowski, Weyers, & Strack, 2009). In the study by Topolinski et al. (2009), the manipulation of processing fluency was realized via the presentation of coherent and incoherent word triads. With the aid of facial electromyography (fEMG), after reading coherent in comparison to incoherent word triads, (a) a stronger activity of the zygomaticus major (smiling muscle), which indicates increased positive affect, (b) a relaxation of the corrugator supercilia (frowning muscle), which indicates decreased negative affect, and (c) a relaxation of the frontalis (forehead muscle), which indicates increased familiarity, could be observed. Based on a comprehensive review of the fluency and affect literature, Topolinski and Strack (e.g., 2009) developed the fluency-affect model of intuition. This model argues that unexpectedly high processing fluency triggers a subtle and short positive affect, which is then used for intuitive judgments (Topolinski & Strack, 2009). Topolinski (2009) suggests that this fluency-affect approach, which was found to be associated with intuitions in the context of semantic coherence, might also play a role for other intuitive judgments that are linked to fluency. Based on these findings, one could assume that fluency-based truth effects may have an essential affective component. Unkelbach, Bayer, Alves, Koch and Stahl (2011) tested this idea by manipulating fluency via repetition and valence via the presentation of positive and negative information. The authors found that positivity had an effect on the perceived truthfulness, but it did not moderate the truth effect caused by repetition. They thus concluded that positive valence does not play a central role in the repetition-based truth effect. However, the authors themselves pointed out that this interpretation is preliminary because it relies entirely on null results (Unkelbach et al., 2011). In an attempt to directly investigate potential interactive effects between affective states and processing fluency on the truth effect, Koch and Forgas (2012) manipulated mood states between subjects via film clips that had a neutral, positive, or negative content prior to a truth effect experiment. Fluency was manipulated by presenting statements of varying readability (with high vs. low visual contrast). The authors hypothesized—based on the assimilative versus accommodative processing model by Bless and Fiedler (2006)—that a positive (negative) mood state should promote (suppress) the reliance on perceived processing fluency for truth judgments. Partially confirming this hypothesis, Koch and Forgas (2012) found a truth effect in the neutral but not in the negative mood condition. Critically viewed, if a positive mood state promoted the reliance on the perceived processing fluency, a stronger truth effect would furthermore be expected in the positive as compared to the neutral mood condition. However, Koch and Forgas’ (2012) descriptive pattern of results rather suggests that the truth effect was also reduced in the positive mood condition. A speculative explanation for this pattern would be that, in a consistently positive mood state, the positive affect induced by high processing fluency may be perceived less clearly, which could in turn reduce the truth effect.

Consequently, whereas the influence of positive affective states on the truth effect remains unclear, the findings indicate that negative mood reduces the truth effect. Somewhat contrary, Hilbig (2009) found that negativity fostered a truth bias: If new information was framed negatively rather than positively, participants were more likely to judge them as true. This effect was replicated several times (Hilbig, 2012a, b). Jaffé and Greifeneder (2019), however, underline in their recent work the importance of expectations in the context of this negativity bias. Based on their studies, they suggest that expectations and framing together increase believability. More precisely, in the case of negatively framed statements, they assume that it is important that the message is less negative than expected.

Taken all together, the interactions of cognitive and affective influences on truth judgments are not yet completely understood. However, it has consistently been demonstrated that fluency experiences have an essential affective component and further that the positive affect, when experiencing relatively high processing fluency, plays an important role in underlying decision-making processes of various fluency-based phenomena (e.g., in respect of visual coherence, semantic coherence, and grammaticality; see Topolinski & Strack, 2009). The fluency-affect model of intuition by Topolinski and Strack (2009) offers a theoretical embedding of those findings and may also provide a theoretical framework for the role of affect in the context of the truth effect. Indeed, the fluency-affect model of intuition may explain some inconsistencies between previous theoretical assumptions and diverse findings in this area. For example, in contrast to the hypothesis that a positive mood state promoted the reliance on perceived processing fluency, Koch and Forgas’ (2012) descriptive pattern of results rather suggests that the truth effect was reduced not only in negative mood but also in the positive mood condition. An explanation for this observation could be that, in a consistently positive mood state, the positive affect induced by high processing fluency may be perceived less clearly, which could in turn reduce the truth effect.

Taking the broad fluency-affect literature as well as previous findings in the context of the truth effect with their various forms of fluency and affect manipulations into account, it seems crucial to reopen the question of whether an affective component of the processing fluency experience plays a decisive role in the mechanisms underlying the truth effect. In doing so, particular attention should be paid to the choice of the affect manipulation form to specifically test whether the mechanism underlying the fluency-based truth effect has a substantial affective component. For this purpose, we conducted two experiments. In Experiment 1, we presented new and repeated statements, which participants had to judge as being true or false. These truth ratings were collected 10 min and 1 week after an exposure phase, in which half of the statements had already been presented. Additionally, we manipulated short-term affective states within participants, but independently from the content of the statements, by briefly presenting positive, negative, or neutral faces as primes prior to each truth judgment. Based on the literature reviewed above, we hypothesized that both high fluency induced via repetition and positive affect induced via priming increase the likelihood of judging a statement as true. We further expected the truth effect to be reduced by negative primes, as far as the positive affect, associated with a fluency experience, is driving the fluency-based truth effect. In Experiment 2, we again manipulated fluency via repetition, but not affective states. Instead, we used an affective misattribution procedure, in which one group of participants received the bogus information that subliminal primes, which were presented between statements, could influence their short-term affective states. If the fluency-based truth effect occurs because fluency experiences trigger positive affective reactions, participants, who were provided with an alternative source for their affective reactions to the fluency experience, should show a diminished truth effect.

Our second research aim was to investigate the extent to which individual differences play a role for the truth effect. Previous experiments have shown that not all individuals are equally susceptible to the truth effect (Nadarevic, 2010). However, results from studies addressing possible dispositional variables in this context are rather inconsistent. Arkes et al. (1991) as well as Boehm (1994) investigated whether need for cognition (NFC) can account for individual differences in the magnitude of the truth effect and found no supporting evidence. Likewise, Newman, Jalbert, Schwarz, and Ly (2020) investigated whether NFC moderates the truth effect and found that experimental instructions have an important influence on the relationship between NFC and the truth effect. While they observed an effect of NFC on the size of the truth effect when participants did not receive information about the truth content of the statements in the exposure phase, the explicit information that true and false statements are presented during the exposure phase diminished this effect. Kim (2002) investigated whether the truth effect depends on a person's skepticism and obtained ambiguous results. Recent findings by DiFonzo, Beckstead, Stupak, and Walders (2016) suggest that the truth effect is moderated marginally by skepticism. Sundar, Kardes, and Wright (2015) examined the impact of repetitive health messages as well as the sensitivity to fluency in the context of the truth effect and demonstrated that persons with a high need for affect (motivation to avoid or approach situations that induce emotions) show an enhanced susceptibility to the illusory truth effect (Study 1).

To contribute to the research in this area, we planned to additionally assess the *preference for deliberation* (PD), *preference for intuition* (PI), and *need for cognitive closure* (NCC) in both experiments. There is an increasing number of two-process models in the literature that compare the intuitive and automatic processing (also called system 1) with an analytical and conscious processing (also called system 2; see Stanovich & West, 2000, and Evans & Stanovich, 2013, for overviews). In line with this general idea, results by Garcia-Marques et al. (2016) showed that the repetition-based truth effect is reduced when participants are motivated and have sufficient cognitive resources available; findings indicating that the truth effect is enhanced by conditions which are considered favorable for system 1 processing. Likewise, it is known that people differ in their preferences for making decisions deliberately or intuitively, and that these different strategies can be attributed to different processing systems (Betsch, 2005). According to Betsch (2005), people with a high preference for deliberation base their judgments more on their cognitive evaluations, while intuitive people base them more on immediately available affects, and—in general—tend to make faster decisions than deliberate individuals. Consequently, we assume that people with high PI scores are more likely to base their truth judgments on immediately available affects and instantly perceived fluency (i.e., that they show larger truth effects). For people scoring high on PD, opposing findings are to be expected. It has further been discussed that NCC may affect the size of the truth effect (Nadarevic, 2010). People with a strong NCC prefer unambiguous situations and make quick decisions with a high subjective confidence (Schlink & Walther, 2007). Consequently, people with a high NCC should be more likely to base their truth judgments on instantly perceived fluency as well as on immediately available affects. However, to the best of our knowledge, these ideas have only been addressed once in recent studies by De keersmaecker et al. (2020). In these studies, no effects were found in the context of the mentioned dispositional variables and the truth effect. However, these findings also have some limitations we will discuss in the General Discussion section.

In the following descriptions of the experiments we report all measures, experimental conditions and data exclusions that were applied and describe how the sample sizes were determined.

## Experiment 1

Participants of this experiment had to judge the truthfulness of different statements. We systematically manipulated fluency of the statements by presenting some of the statements during a preceding task. While making the judgments, short-term affective states were induced via briefly presented positive, negative, or neutral primes. Both fluency and affect were manipulated within subjects.

The implementation of the affective priming was modeled closely following Topolinski and Strack’s (2009) procedure, and was applied between statement presentation and truth judgment, to ensure that the affect manipulation would take influence on the judgment directly rather than on the initial processing of the statement. For the affect induction, a positive, negative or neutral prime (17 ms) in the form of a happy, sad, or neutral facial expression, each masked by a neutral facial expression (350 ms), was presented between every statement presentation and prompt for the truth judgment. The very short priming presentation was applied to make sure that participants were not able to fully discern the source of their short-term affective states, which in turn should facilitate the possibility of a misattribution process. This method has already been used in former studies which rendered empirical support for its effectiveness in causing short-term affective changes (e.g., Topolinski & Strack, 2009). Using a fEMG, Dimberg, Thunberg and Elmehed (2000) further demonstrated that such a backward masking technique (subliminal exposure to emotional target faces, masked with neutral faces) causes facial muscle reactions which correspond to the emotional expression of the subliminally presented stimulus.

Nadarevic and Erdfelder (2014) showed that the length of the retention interval and the form of the first statement exposure moderate the truth effect. They found no truth effect when using the typical judgment design with a retention interval length of ten minutes. However, they observed a truth effect when the statements did not have to be judged according to their truthfulness in the first phase of the experiment, or when a retention interval of one week was used instead of ten minutes (Nadarevic & Erdfelder, 2014). These findings suggest that memory-based processes have a stronger impact on truth judgments given after a short retention interval, whereas the perceived processing fluency is probably even more the central component in the mechanism underlying the truth effect after a longer interval (for further research results corresponding to this assumption, see also Garcia-Marques et al., 2015; Nadarevic, Plier, Thielmann, & Darancó, 2018). Therefore, the length of the retention interval may have a significant influence on the determinants we investigate in the context of the truth effect (fluency, fluency-triggered positive affect, and dispositional differences). Consequently, we realized two testing sessions, that is, truth judgments were queried both ten minutes after the first exposure and again one week later. Additionally, we did not ask participants to provide truth judgments in the first statement exposure phase and employed a semantic categorization task for the statements instead.

### Method

#### Participants and design

In total, 109 student participants were recruited at Heidelberg University with the recruitment software hRoot (Bock, Baetge, & Nicklisch, 2014). We excluded data from one person who was not a native speaker of German, from three participants who did not attend the second testing session, from one person with visual impairment, and from seven participants who did not follow task instructions or behaved inappropriately during testing (e.g., talked to themselves, approached the experimenter in an offensive manner). The remaining 97 participants were between 18 and 31 years old (*M* = 22.15, *SD* = 3.15) and 79% were female.^2^ The majority of participants (79%) were non-psychology students. Participants received 10 Euros (approximately 10.9 US$) or course credit for their participation.

The design comprised the three within-subject factors *repetition status* (new vs. repeated), *valence of prime* (positive vs. neutral vs. negative), and *retention interval* (ten minutes vs. one week).

#### Material

The main statement material consisted of 120 statements in German (four statement sets). Eight additional statements served as a buffer against possible primacy and recency effects (the first and last four statements within the exposure phase). Unkelbach and Stahl (2009) showed that—in the case of truth judgments—a reliance on fluency is especially likely when there is uncertainty about the actual truth of a statement. We therefore selected statements from Nadarevic’s (2010) dissertation thesis that had been carefully pretested to be difficult enough and had produced a reliable truth effect in previous studies (Nadarevic, 2010). Furthermore, we selected only statements that we considered to be affectively neutral in terms of content, as their content should not trigger any affective reactions. Likewise, we ensured that all statements had a similar length so that their individual processing takes a similar amount of time. The selected statements were divided into four sets (A–D) with 15 true and 15 false statements each. Truth ratings (as provided by Nadarevic, 2010) from all four sets had comparable means and standard deviations (set A: *M* = 4.06, *SD* = 1.20; set B: *M* = 4.06, *SD* = 1.18; set C: *M* = 4.05, *SD* = 1.18; set D: *M* = 4.05, *SD* = 1.20; rated on a seven-point scale). In each test session two sets of statements were used, one of which had already been presented to the participants in the exposure phase. The assignment of the four sets to the different phases of the experiment was counter-balanced across participants.

For the affective priming, two photo sets with 30 Caucasian females and 30 Caucasian males were formed. In each judgment phase 30 repeated and 30 new statements with 10 × positive prime (smiling face), 10 × neutral prime (neutral facial expression), 10 × negative prime (sad face) each, and corresponding mask (neutral facial expression), were presented. The neutral masks were portraits that belonged to the same individuals as those of the previously presented primes. Therefore, both assembled photo sets contained a total of 100 different photos. To obtain enough photos, images from the *Karolinska Directed Emotional Faces* database (KDEF, Lundqvist, Flykt, & Öhman, 1998) were used for one set. For the other set, images from the *Radboud Faces Database* (RaFD, Langner et al., 2010) and *Warsaw Set of Emotional Facial Expression Pictures* (WSEFEP, Olszanowski et al., 2015) were used. The assignment of photo sets to testing sessions was counter-balanced across subjects.

To assess the individual *need for cognitive closure*, we used the German short scale from Schlink and Walther (2007). The NCC scale consists of 16 items. For each item, participants indicate how strongly the respective statement expresses their personal attitude, opinion and experience, using a six-point Likert scale ranging from 1 (completely disagree) to 6 (completely agree). Exemplary items are “In general, I do not look for alternative solutions to problems for which I already have a solution.” or “Looking at a problem from different perspectives only leads to confusion.” (note that these are English translations of the original German items). Cronbach's alpha (*α* = 0.82) indicated a good internal consistency.

The *preference for making decisions deliberately vs. intuitively* was assessed using the Preference for Intuition and Deliberation Scale (PID, Betsch, 2004). The scale consists of 18 items, nine items to measure the preference for intuition and nine items to measure the preference for deliberation. For each item, participants indicate how strongly the respective statement expresses their strategy, with which they generally make decisions, using a five-point Likert scale ranging from 1 (completely disagree) to 5 (completely agree). Example items are “Emotions play an important role in my decision making.” or “In most decisions, it makes sense to rely entirely on your feelings.” for the preference for intuition, and “Before I decide, I first break down the hard facts and details.”, or “Most of the time I think carefully, before I make decisions.”, for the preference for deliberation (translations from the original German items). Cronbach's alpha values indicated acceptable internal consistencies of the scales for preference for intuition (*α* = 0.71) and preference for deliberation (*α* = 0.80).

#### Procedure

Up to six participants took part in one experimental session. After they signed a consent form and provided basic demographic information, the computer experiment started. During the first phase of the experiment, 68 statements were presented one-by-one, and participants were instructed to classify the (true and false) statements into predetermined categories, namely (1) Geography, (2) Biology, (3) Politics & History, (4) Science, (5) Entertainment and (6) Others. The first and last four statements were the same for all participants and served as a buffer against possible primacy and recency effects. The 60 statements in between were presented in random order and were taken from two statement sets with 15 true and 15 false statements each. Each trial started with the presentation of a fixation cross with a random duration between 250 and 750 ms. The time of the fixation-cross presentation varied to counteract the development of expectations about the presentation time and to keep participants on task. The following statement was shown for 3500 ms in the center of the screen.^3^ After the presentation of each statement, participants were asked to classify the previously presented statement into one of the six knowledge categories by pressing one of the number keys. There was no time limit, although the participants were asked to make their decisions as quickly as possible, without making unnecessary mistakes to encourage them to work swiftly and carefully on the task.^4^

After a ten-minute retention interval, in which the subjects worked on a non-verbal filler task, the second phase of the experiment (i.e., first judgment phase) started. During this phase, 30 new and 30 repeated statements were presented one-by-one in random order, and participants were instructed to judge whether each statement was true or false. Participants were additionally informed that neutral portrait photos would be displayed after each statement to inform them that they will shortly be asked to give their truth judgment. Thirty of the statements (repeated statements) originated from one of the statement sets of the exposure phase, while the 30 new statements originated from one of the not yet used statement sets. Again, a fixation cross was shown for 250–750 ms before the presentation of the statement. After 3500 ms, the statement was removed from the screen and replaced by a face with happy, sad, or neutral expression that was presented for 17 ms in the center of the screen. The emotional primes were immediately masked by a photo of the same individual showing a neutral expression, which remained on the screen for 350 ms. The presented emotional expressions were randomly determined, ensuring that each of the three expressions occurred equally often and that half of all faces of each type were female. After the neutral face disappeared, participants had to judge the truth of the previously presented statement on a dichotomous scale (“true” vs. “false”) by pressing the “W” key (“wahr”, German for true) or “F” key (“falsch”, German for false). Thereafter, participants had to rate the confidence with which they had made the truth judgment, using a six-point scale from 1 (very uncertain) to 6 (very certain) by pressing one of the upper number keys (1–6). There was no time limit either for the truth judgments or the confidence ratings. As in the exposure phase, participants were instructed to make their ratings as fast as possible, without making evitable mistakes.

In the second session, which was always scheduled exactly 7 days after the first session, participants attended a second judgment phase whose procedure was identical to the first judgment phase, with the exception that new material was used. That is, the final un-used set of statements was presented intermixed with those statements from the exposure phase that had not been used during the first judgment phase. After the second judgment phase, participants filled in paper–pencil versions of the NCC and the PID scale. Subsequently, they were asked to state (a) whether they had any hypotheses regarding the aim of this study, (b) whether they had informed themselves about the truth content of statements occurring in the experiment between sessions, and (c) whether they had noticed any optical abnormalities between the statement presentation and the presentation of the neutral portraits. When a question was answered with "yes", the subjects should accordingly state which hypotheses they had regarding the aim of this study (a), about which statements of the experiment they had informed themselves (b), or which optical abnormalities they had noticed (c).

### Results

A multilevel modeling approach was used for all investigations, which allows the simultaneous consideration of state (level-1) and trait (level-2) predictors as well as within- and cross-level interactions as predictors of truth and confidence ratings.

For the analyses, the level-1 predictors were dummy coded. For the level-1 predictor *repetition status*, the new (disfluent) statements served as reference group. The level-1 predictor *prime valence* had three categories (positive/neutral/negative), so that two dummy-coded variables were formed. In this context, the condition with neutral affective primes served as reference condition and the two dummy-coded variables were termed *positive valence* and *negative valence*. This coding was used because it allowed testing our hypotheses in the most efficient manner; especially, the higher probability to judge a statement as true when it is repeated instead of new (i.e., truth effect), the influence of positive affect on truth judgments for new statements, as well as the impact of negative affect and different dispositional variables (NCC, PI, & PD) on the truth effect.

The level-2 predictors *need for cognitive closure*, *preference for intuition,* and *preference for deliberation* were grand-mean centered. Results by Betsch (2004) indicate that there are slightly negative correlations between the trait variables *need for cognitive closure (NCC)* and *preference for intuition (PI)* as well as between *preference for intuition (PI)* and *preference for deliberation (PD)*. Correlations between these variables were calculated. However, as can be seen in Table 1, no significant correlations between NCC and PI, between NCC and PD, or between PI and PD were found (in all cases *p* > 0.05).

**Table 1:** Pearson correlations, Cronbach’s Alpha (in parentheses), Means and Standard Deviations for scores on the PI-scale, PD-scale and NCC scale (Experiment 1)

Variables	1	2	3	*M*	*SD*
1. PI	(0.71)			3.50	0.54
2. PD	− 0.112	(0.80)		3.84	0.63
3. NCC	− 0.103	0.163	(0.82)	3.38	0.64

*N* = 97

*PI* preference for intuition, *PD* preference for deliberation, *NCC* need for cognitive closure

For all correlations: *p* > 0.05

As a manipulation check, we tested whether repetition status (a source of conceptual and perceptual processing fluency) had an impact on the response times for truth judgments.^5^ For this analysis, the predictors *repetition status*, *judgment phase* (coded − 0.5 for the first judgment phase and + 0.5 for the second judgment phase) as well as the interaction involving these variables were included into the model. Results showed a significant main effect for repetition status (*b* = − 0.134, *p* < 0.001) and judgment phase (*b* = − 0.122, *p* < 0.001), as well as an interaction between repetition status and judgment phase (*b* = 0.057, *p* = 0.016). These results indicate that the participants made their truth ratings more quickly when the statement was repeated and generally rated the truthfulness of new statements faster during the second judgment phase. The length of the time interval between the first exposure and the later judgment sessions influenced the effect of repetition status (the effect of repetition status was reduced at the longer interval).

#### Truth judgments

The analyses were performed with the statistical software R (version 3.4.3) using the lme4 package (Bates, Mächler, Bolker, & Walker, 2015). Truth ratings were analyzed with a generalized linear mixed model based on maximum likelihood (Laplace approximation). To account for the dichotomous nature of the criterion (true, false), a logit link function was applied. Therefore, the logit of the conditional probability that the outcome variable truth judgment equals one (true) over the probability that the outcome variable equals zero (false) was predicted. All models included random intercepts for subjects and items. Because convergence problems arose when adding random slopes to the model, all predictors were treated as fixed effects.

In a first step—to consider the possible effects of the retention interval length—the level-1 predictors *repetition status*, *positive valence*, *negative valence* and the dichotomous variable *judgment phase* (coded − 0.5 for the first judgment phase and + 0.5 for the second judgment phase) as well as all interactions involving these variables were entered into the model.^6^ The predicted main effect of repetition status (*b* = 0.744, *p* < 0.001) was found. Results also show that judgment phase interacted with negative valence (*b* = 0.287, *p* = 0.035) and with repetition status (*b* = − 0.423, *p* = 0.003). Furthermore, a three-way interaction between repetition status, negative valence and judgment phase (*b* = − 0.456, *p* = 0.021) was found. These findings imply that the length of the time interval between the first exposure and the later judgment sessions influenced the experimental manipulations. Consequently, all following analyses were conducted separately for the first and second judgment phase.

We also checked for possible material effects induced by the different photo sets, for differences between participants who were aware of the study aim and those who were not, and for differences between participants who were aware of the affective priming and those who were not. However, the present pattern of results was not significantly affected by either of these factors (see Appendix D).

#### Truth judgments after the ten-minute interval

In a first model, referred to as Model 1a,^7^ the level-1 predictors repetition status, positive valence, negative valence as well as all interactions involving these variables were included. Results reveal only a significant main effect for repetition status (*b* = 0.973, *p* < 0.001), indicating an increased probability of truth ratings for repeated compared to new statements (in the case of neutral priming). The odds ratio (*OR*) is 2.6, indicating that the response "true" (vs. "false") was 2.6 times more likely when a statement was repeated. Thus, we replicated the basic truth effect, that people are more likely to judge a repeated (fluent) statement as true as compared to a new (disfluent) statement. No significant effects were obtained for positive valence (*b* = 0.064, *p* = 0.507) or for negative valence (*b* = − 0.096, *p* = 0.317), and no significant interaction between repetition status and positive valence (*b* = − 0.062, *p* = 0.663) or between repetition status and negative valence (*b* = 0.131, *p* = 0.361) was found. Table 2 shows all results.

**Table 2:** Multilevel logistic modeling results for the prediction of "true" responses after the ten-minute interval (Experiment 1)

	Fixed Effects							
	Model 1a				Model 2a			
	*b*	*SE*	*z*	*p*	*b*	*SE*	*z*	*p*
Intercept	0.029	0.095	0.310	0.757	0.020	0.076	0.262	0.793
Repetition status	0.973	0.101	9.597	< 0.001***	1.003	0.059	16.994	< 0.001***
Valence (pos.)	0.064	0.096	0.663	0.507	–	–	–	–
Valence (neg.)	– 0.096	0.096	– 1.000	0.317	–	–	–	–
R.S. × Valence (pos.)	– 0.062	0.143	– 0.435	0.663	–	–	–	–
R.S. × Valence (neg.)	0.131	0.143	0.914	0.361	**–**	**–**	**–**	**–**
PI	–	–	–	–	0.129	0.111	1.169	0.242
PD	–	–	–	–	0.180	0.096	1.867	0.062
NCC	–	–	–	–	0.009	0.093	0.094	0.925
Repetition Status × PI	–	–	–	–	– 0.001	0.110	– 0.010	0.992
Repetition Status × PD	–	–	–	–	– 0.353	0.096	– 3.660	< 0.001***
Repetition Status × NCC	–	–	–	–	0.371	0.097	3.824	< 0.001***

*N* = 97. ***p < 0.001

*R.S.*  repetition status, *pos.*  positive, *neg.*  negative, *PI* preference for intuition, *PD* preference for deliberation, *NCC* need for cognitive closure

In the second model (Model 2a), *repetition status* was included as the only level-1 predictor and furthermore the level-2 predictors *preference for intuition*, *preference for deliberation* and *need for cognitive closure* as well as all cross-level interactions involving these variables were added (Table 2 displays all results). A decrease in the Akaike information criterion (AIC) and Bayesian information criterion (BIC) indicated an improved model fit for Model 2a compared to Model 1a (Model 1a: AIC = 7251.5 & BIC = 7304.8; Model 2a: AIC = 7228.6 & BIC = 7295.3), suggesting that the individual difference variables explained substantial variance in truth judgments.

**Fig. 1:**
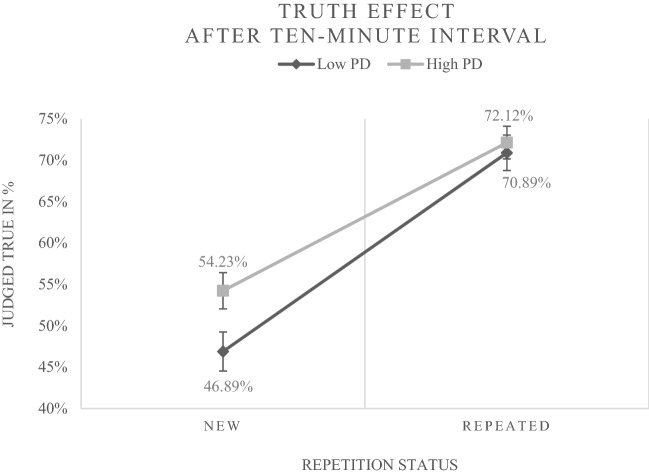
The figure displays the percentage of new (disfluent) and repeated (fluent) statements judged true by persons with a high vs. low preference for deliberation (PD) after the ten-minute interval in Experiment 1 (data from trials with neutral primes). For a clear illustration a median split for PD was accomplished (*Mdn* = 3.89). Error bars represent standard errors

In addition to the main effect of repetition status (*b* = 1.003, *p* < 0.001; *OR* = 2.7), the predicted cross-level interaction of repetition status and preference for deliberation was revealed (*b* = − 0.353, *p* < 0.001). This finding implies that the effect of repetition status was reduced for participants with a higher preference for deliberation. Figure 1 illustrates the observed frequencies underlying this result.

**Fig. 2:**
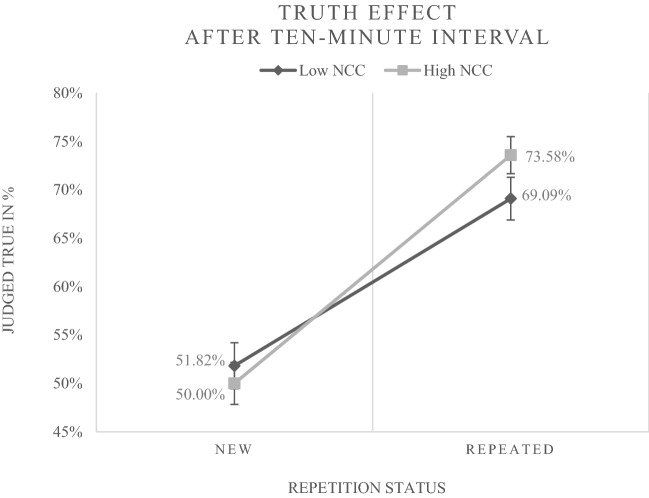
The figure displays the percentages of new (disfluent) and repeated (fluent) statements judged true by persons with a high vs. low need for cognitive closure (NCC) after the ten-minute interval in Experiment 1 (data from trials with neutral primes). For a clear illustration a median split for NCC was accomplished (*Mdn* = 3.38). Error bars represent standard errors

The predicted cross-level interaction between *repetition status* and *need for cognitive closure* was also significant (*b* = 0.371, *p* < 0.001), suggesting that the truth effect (i.e., the effect of repetition status) was stronger for people with a higher need for cognitive closure. Figure 2 illustrates the frequencies underlying this effect. The expected cross-level interaction between *repetition status* and *preference for intuition* was not significant (*b* = − 0.001, *p* = 0.992).

#### Truth judgments after the one-week interval

**Fig. 3:**
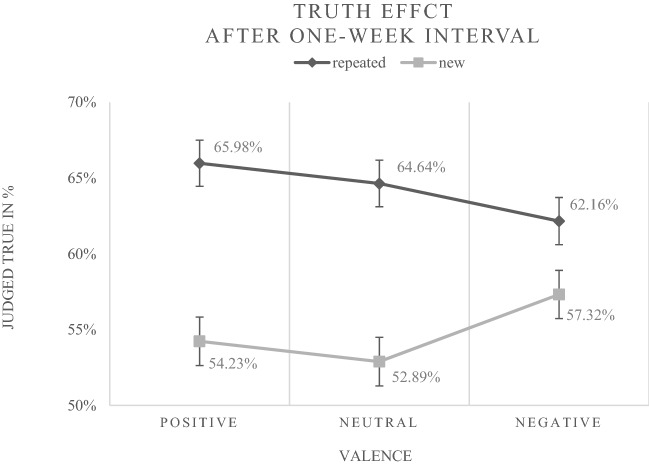
Percentages of "true" judgments for new (disfluent) and repeated (fluent) statements were represented as a function of prime valence after the one-week interval in Experiment 1. Error bars represent standard errors

Again, the level-1 predictors *repetition status*, *positive valence*, *negative valence* as well as all interactions involving these variables were included in a first model, referred to as Model 1b (see Table 3). A repetition-induced truth effect was also found after the one-week interval (*b* = 0.522, *p* < 0.001; *OR* = 1.7), indicating that the response "true" (vs. "false") was 1.7 times more likely when a statement was repeated (in the case of neutral priming). Furthermore, an interaction between negative valence and repetition status was found (*b* = − 0.305, *p* = 0.027). The negative value of the regression weight implies that the truth effect was decreased by negative primes (see Fig. 3 for an illustration of the frequencies underlying this finding).

**Table 3:** Multilevel logistic modeling results for the prediction of "true" responses after the one-week interval (Experiment 1)

	Fixed Effects							
	Model 1b				Model 2b			
	*b*	*SE*	*z*	*p*	*b*	*SE*	*z*	*p*
Intercept	0.141	0.091	1.546	0.122	0.142	0.091	1.555	0.120
Repetition Status	0.522	0.098	5.347	< 0.001 ***	0.522	0.098	5.327	< 0.001 ***
Valence (positive)	0.044	0.096	0.462	0.644	0.044	0.096	0.454	0.650
Valence (negative)	0.172	0.096	1.791	0.073	0.172	0.097	1.779	0.075
R.S. × Valence (pos.)	0.030	0.139	0.219	0.827	0.031	0.139	0.225	0.822
R.S. × Valence (neg.)	– 0.305	0.138	– 2.210	0.027 *	– 0.302	0.138	– 2.179	0.029 *
PI	–	–	–	–	– 0.260	0.148	– 1.761	0.078
PD	–	–	–	–	0.109	0.128	0.852	0.394
NCC	–	–	–	–	0.187	0.124	1.507	0.132
Repetition Status × PI	–	–	–	–	0.262	0.186	1.409	0.159
Valence (pos.) × PI	–	–	–	–	0.539	0.183	2.947	0.003 **
Valence (neg.) × PI	–	–	–	–	0.374	0.183	2.048	0.041 *
Repetition Status × PD	–	–	–	–	– 0.028	0.161	– 0.172	0.864
Valence (pos.) × PD	–	–	–	–	0.072	0.158	0.455	0.649
Valence (neg.) × PD	–	–	–	–	– 0.134	0.158	– 0.849	0.396
Repetition Status × NCC	–	–	–	–	– 0.214	0.156	– 1.373	0.170
Valence (pos.) × NCC	–	–	–	–	– 0.274	0.153	– 1.788	0.074
Valence (neg.) × NCC	–	–	–	–	– 0.135	0.154	– 0.872	0.383
R.S. × Valence (pos.) × PI	–	–	–	–	– 0.513	0.264	– 1.946	0.052
R.S. × Valence (neg.) × PI	–	–	–	–	– 0.412	0.262	– 1.571	0.116
R.S. × Valence (pos.) × PD	–	–	–	–	– 0.117	0.227	– 0.517	0.605
R.S. × Valence (neg.) × PD	–	–	–	–	– 0.154	0.227	– 0.680	0.497
R.S. × Valence (pos.) × NCC	–	–	–	–	0.302	0.221	1.366	0.172
R.S. × Valence (neg.) × NCC	–	–	–	–	0.389	0.221	1.758	0.079

*N* = 97

*R.S.*  repetition status, *pos.*  positive, *neg.*  negative, *PI* preference for intuition, *PD* preference for deliberation, *NCC* need for cognitive closure

*p < 0.05; **p < 0.01; ***p < 0.001

In a next step, the level-1 predictors *repetition status*, *positive valence*, *negative valence* as well as all interactions involving these variables, the level-2 predictors *preference for intuition*, *preference for deliberation* and *need for cognitive closure* as well as all cross-level interactions involving these variables were included in Model 2b.^8^ Increases in both AIC and BIC indicated a deteriorated model fit for Model 2b compared to Model 1b (Model 1b: AIC = 7573.1 & BIC = 7626.4; Model 2b: AIC = 7585.4 & BIC = 7758.8), which is why results of Model 2b should be considered with caution.

**Fig. 4:**
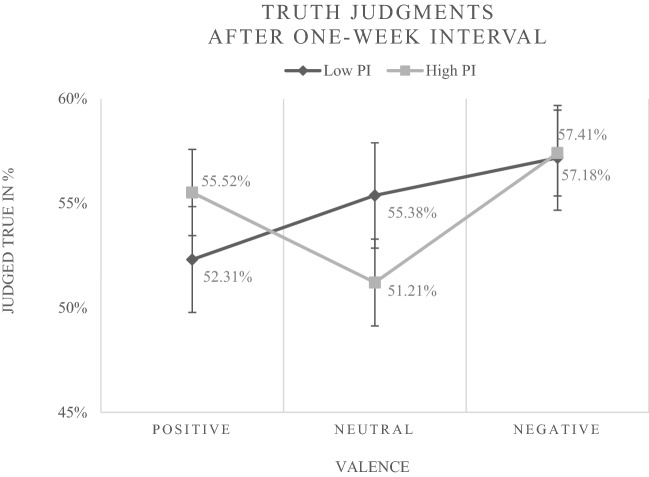
Percentages of new statements judged true in the case of positive, neutral and negative affective priming by individuals with high vs. low preference for intuition (PI) after the one-week interval in Experiment 1. For a clear illustration a median split for PI was accomplished (*Mdn* = 3.44). Error bars represent standard errors

As evident from Table 3, in addition to the main effect of *repetition status* (*b* = 0.522, *p* < 0.001; *OR* = 1.7), and the within-level interaction between *repetition status* and *negative valence* (*b* = – 0.302, *p* = 0.029), the predicted cross-level interaction between *positive valence* and *preference for intuition* (*b* = 0.539, *p* = 0.003) as well as the predicted cross-level interaction between *negative valence* and *preference for intuition* (*b* = 0.374, *p* = 0.041) significantly influenced the truth judgments. According to this finding, the effect of positive and negative valence (the higher probability of judging a new statement as true in the case of positive or negative priming compared to neutral priming) was stronger for people with a higher preference for intuition. Figure 4 illustrates the observed frequencies underlying this finding.

#### Discussion

Experiment 1 revealed a repetition-based truth effect, and demonstrated that this effect was stronger, when the repetition interval was short (10 min) rather than long (1 week). The findings also show that the truth effect was reduced by negative affective primes (after the one-week interval), as well as that the effect of repetition was increased by need for cognitive closure, and decreased by preference for deliberation, after the ten-minute interval. Preference for intuition had, against our hypotheses, no influence on the truth effect's size. Likewise, we found no main effect of positive valence (i.e., the higher probability of judging a new statement as true in the case of positive priming compared to neutral priming).

One central finding of Experiment 1 was that the truth effect was reduced when statements were proceeded by a negative prime. This finding is in line with our hypothesis that the positive affect, which has been shown to accompany fluency experiences, is critical to the occurrence of the fluency-related truth effect. However, this interaction was only found after the longer time interval. Possibly, a relatively strong fluency experience is the reason why the truth effect was not reduced by the rather subtle influence of subliminal negative primes after the short retention interval (10 min). To provide further evidence in this respect and to replicate the significant individual differences in the extent of the truth effect, we conducted a second experiment.

## Experiment 2

Experiment 2 aimed to replicate and extend the findings from Experiment 1. To this end, we again manipulated fluency via the presentation of new and repeated statements that had to be judged as true or false (half of the statements were presented during a preceding semantic categorization task). As in Experiment 1 we realized two judgment phases (one 10 min after the first exposure, and another 1 week later). However, no affective primes were included in Experiment 2. Instead, we created two experimental groups: One group was provided with the bogus information that subliminal affective primes would be presented during the judgment task, and that these primes might change their affective experiences; the other group did not receive any information about potential affect inductions that may occur during the task. This manipulation aimed to provide an alternative explanation for potential changes in affective states associated with a repetition-based fluency experience in the former group. Therefore, we hypothesized that the fluency-based truth effect would be reduced in the former compared to the latter group if this effect is driven by the increase in positive affect associated with a fluency experience.

### Method

#### Participants and design

In total, 86 student participants were recruited at Heidelberg University with the recruitment software hRoot (Bock et al., 2014). We excluded data from one person who was not a native speaker of German, from five participants who did not attend the second testing session, and from five participants due to experimenter errors (i.e., incorrect condition assignments). The remaining 75 participants were between 18 and 32 years old (*M* = 21.56, *SD* = 2.95), 68% were female.^9^ The majority of participants (77%) were non-psychology students. Participants received 10 Euros (approximately 10.9 US$) or course credit for their participation.

The design comprised the between-subject factor *affect attribution* (control group vs. affect attribution group) as well as the two within-subject factors *repetition status* (new vs. repeated), and *retention interval* (ten minutes vs. one week).

#### Material

We used the same 120 statements as in Experiment 1, which were again divided into four sets (Set A to D, with 15 true and 15 false statements each). Twelve additional statements served as a buffer against possible primacy and recency effects (the first and last six statements within the exposure phase). The assignment of the four sets to the different phases of the experiment was counter-balanced across participants.

To assess the individual *need for cognitive closure*, we again used the German short scale by Schlink and Walther (2007). Cronbach's alpha (*α* = 0.72) indicated an acceptable internal consistency of this short scale. The preference for making decisions deliberately versus intuitively was assessed using the *Preference for Intuition and Deliberation Scale* (PID, Betsch, 2004). Internal consistencies of the scales for preference for intuition (*α* = 0.75) and preference for deliberation (*α* = 0.80) were acceptable.

#### Procedure

Up to seven participants took part in each session. The basic procedure was identical to the one of Experiment 1 (i.e., the first session included the exposure phase, a filler task (10 min) and the first judgment phase; the second session, which followed exactly seven days after the first session, included the second judgment phase and paper-pencil versions of the NCC and the PID scale). However, this time affect was not manipulated and thus no affective pictures were presented. Instead, two experimental groups (a control group vs. an affect attribution group) were realized. Participants in the control group were instructed that they had to judge the truth content of different statements and afterwards rate how confident they were with the respective truth judgment. Participants in the affect attribution group received the additional bogus information that subliminal affective primes would be presented before judgments were made and that these primes could trigger affective reactions during the experiment. The affective priming was described as a manipulation using emotional facial expressions presented very briefly, so that one would probably perceive only a short flickering of the screen. We also pointed out that the primes would not be related to the truth content of the statements in any way. To support this cover story, in both judgment phases a black screen was briefly displayed before each truth judgment (17 ms), which produced a slight flickering of the screen.

### Results

Again, a multilevel modeling approach was used for all investigations.

The categorical predictors *repetition status* and *affect attribution* were dummy coded. In the case of *repetition status* the new (disfluent) statements, and in case of the variable *affect attribution* the control group (participants without any information about an irrelevant source for changes in affective states) served as reference group. This coding was applied because it allowed us to test our hypotheses in the most efficient way; especially, the higher probability of judging a statement to be true when it is repeated rather than new (i.e., truth effect), the impact of an alternative source for changes in short-term affective states and effects of the different dispositional variables (NCC, PI, & PD) on the truth effect. The predictors *need for cognitive closure*, *preference for intuition,* and *preference for deliberation* were grand-mean centered.

Correlations between the variables need for cognitive closure (NCC), preference for intuition (PI), and preference for deliberation (PD) were calculated. As can be seen in Table 4, no significant correlation between NCC and PI, between NCC and PD, or between PI and PD was found (in all cases *p* > 0.05).^10^

**Table 4:** Pearson correlations, Cronbach’s Alpha (in parentheses), Means and Standard Deviations for scores on the PI-scale, PD-scale and NCC-scale (Experiment 2)

Variables	1	2	3	*M*	*SD*
1. PI	(0.75)			3.17	0.63
2. PD	− 0.187	(0.80)		4.10	0.58
3. NCC	0.072	− 0.016	(0.72)	3.40	0.56

*N* = 69

*PI* preference for intuition, *PD* preference for deliberation, *NCC* need for cognitive closure

For all correlations: *p* > 0.05

As a manipulation check, we tested whether repetition status affects the response times for truth judgments.^11^ Therefore, the predictors *repetition status*, *judgment phase* (coded – 0.5 for the first judgment phase and + 0.5 for the second judgment phase) as well as the interaction involving these variables were integrated into the model. Results reveal a significant main effect of repetition status (*b* = – 0.223, *p* < 0.001) and judgment phase (*b* = – 0.089, *p* < 0.001), as well as an interaction between repetition status and judgment phase (*b* = 0.103, *p* < 0.001). The findings show that the participants gave their truth ratings more quickly for repeated statements, and generally rated the truth content of new statements faster during the second judgment phase. The length of the time interval between the first exposure and the later judgment phases influenced the effect of repetition status (a longer interval reduced the effect).

#### Truth judgments

The analyses were performed with the statistical software R (version 3.6.1) using the lme4 package (Bates et al., 2015). A generalized linear mixed model fit by maximum likelihood (Laplace approximation) was used. To account for the dichotomous nature of the criterion (true, false), a logit link function was utilized. All models included random intercepts for subjects and items.

**Fig. 5:**
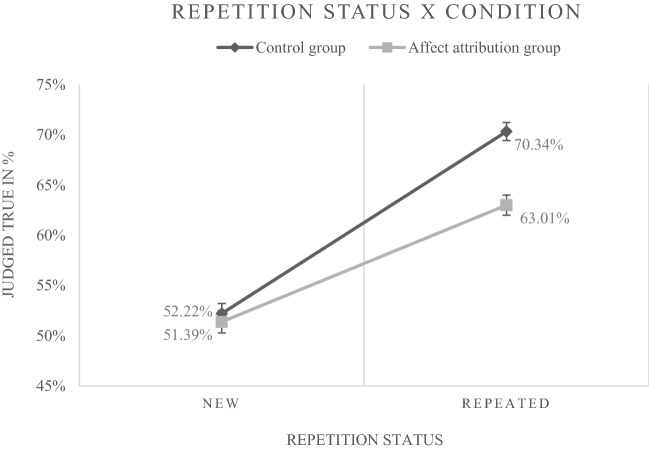
Percentages of "true" judgments for new (disfluent) and repeated (fluent) statements in the control and affect attribution group (Experiment 2). Error bars represent standard errors

In a first model, referred to as Model1a,^12^ the predictors *repetition status*, *affect attribution* and *judgment phase* as well as all interactions involving these variables were included. The predicted main effect of repetition status (*b* = 0.872, *p* < 0.001) was found. The odds ratio (*OR*) is 2.4, indicating that—in the control group—the response "true" (vs. "false") was 2.4 times more likely when a statement was repeated. Likewise, the predicted interaction between repetition status and affect attribution (*b* = – 0.341, *p* < 0.001) was obtained (Fig. 5 illustrates the frequencies underlying this finding). In addition to these effects, results showed a significant main effect for judgment phase (*b* = 0.304, *p* < 0.001), as well as an interaction between judgment phase and repetition status (*b* = – 0.588, *p* < 0.001), indicating a reduced effect of repetition after the longer interval. However, since the interval length does not significantly influence any other effects, we decided against separate analyses for the two judgment phases, but to include the variable judgment phase in all following analyses.^13^ Table 5 shows all results.

**Table 5:** Multilevel logistic modeling results for the prediction of "true" responses (Experiment 2)

	Fixed Effects							
	Model 1a				Model 2a			
	*b*	*SE*	*z*	*p*	*b*	*SE*	*z*	*p*
Intercept	0.118	0.111	1.063	0.288	0.099	0.114	0.869	0.385
Repetition Status	0.872	0.066	13.223	< 0.001***	0.870	0.069	12.672	< 0.001***
Affect Attribution	– 0.049	0.138	– 0.357	0.721	– 0.010	0.144	– 0.070	0.944
Judgment Phase	0.304	0.089	3.417	< 0.001***	0.358	0.093	3.860	< 0.001***
R.S. × Affect Attribution	– 0.341	0.093	– 3.674	< 0.001***	– 0.381	0.097	– 3.915	< 0.001***
R.S. × Judgment Phase	– 0.588	0.131	– 4.481	< 0.001***	– 0.630	0.137	– 4.607	< 0.001***
A.A. × Judgment Phase	– 0.155	0.128	– 1.210	0.226	– 0.223	0.133	– 1.672	0.095
R.S. × A.A. × J. P.	0.295	0.186	1.592	0.111	0.383	0.194	1.977	0.048*
PI	–	–	–	–	0.075	0.118	0.637	0.524
PD	–	–	–	–	– 0.081	0.127	– 0.636	0.525
NCC	–	–	–	–	0.061	0.129	0.475	0.635
Repetition Status × PI	**– **	**– **	**– **	**– **	0.135	0.081	1.668	0.095
Repetition Status × PD	**– **	**– **	**– **	**– **	0.119	0.086	1.392	0.164
Repetition Status × NCC	**– **	**– **	**– **	**– **	0.219	0.088	2.481	0.013*
R.S. × PI × J.P.	**– **	**– **	**– **	**– **	– 0.096	0.117	– 0.820	0.412
R.S. × PD × J.P.	**– **	**– **	**– **	**– **	0.037	0.125	0.293	0.770
R.S. × NCC × J.P.	**– **	**– **	**– **	**– **	– 0.200	0.130	– 1.538	0.124

*N* = 75 (Model 1a); *N* = 69 (Model 2a)

*R.S.* repetition status, *J.P.* judgment phase, *A.A.*  affect attribution, *PI* preference for intuition, *PD* preference for deliberation, *NCC* need for cognitive closure

*p < 0.05; ***p < 0.001

In the second model (2a), in addition to the predictors which were already included in our first model (Model 1a), the variables *preference for intuition*, *preference for deliberation* and *need for cognitive closure*, cross-level interactions involving these and repetition status as well as three-way interactions involving these dispositional variables, repetition status and judgment phase were added. The pattern of results (as reported above) did not change, except that the three-way interaction *repetition status* × *affect attribution* × *judgment phase* now just reached statistical significance (*b* = 0.383, *p* = 0.048), indicating that the interaction between repetition status and affect attribution was reduced after the longer time interval. In addition, the predicted cross-level interaction between repetition status and need for cognitive closure was found (*b* = 0.219, *p* = 0.013), suggesting that the truth effect was stronger for people with a higher need for cognitive closure.^14^ The expected cross-level interaction between repetition status and preference for intuition (*b* = 0.135; *p* = 0.095), as well as between repetition status and preference for deliberation (*b* = 0.119, *p* = 0.164) were not significant. All results are displayed in Table 5.

AIC and BIC increases indicated a deteriorated model fit for the second model compared to the first model (Model 1a^15^: AIC = 10322.2 & BIC = 10392.4; Model 2a: AIC = 10323.3 & BIC = 10456.7).

### Discussion

Results of Experiment 2 replicated the repetition-based truth effect. As in Experiment 1, this truth effect was stronger when the repetition interval was short (10 min vs. 1 week) and was increased by a higher need for cognitive closure. The decrease of the truth effect associated with a higher preference for deliberation could not be replicated. Most important, participants who obtained an alternative explanation for changes in their affective states showed a significantly reduced truth effect. This finding again strongly suggests that an affective mechanism underlies the truth effect.

## General discussion

The central aim of the present research was to investigate the effect of processing fluency and short-term affective states on truth judgments. Additionally, we were interested in the impact of individual differences in preference for intuition, preference for deliberation and need for cognitive closure. Besides the effects on truth judgments, we also investigated how these factors would influence the subjective confidence with which truth judgments are made (see Appendix A–C).

In two studies, we observed a repetition-based truth effect: Participants were more likely to judge a repeatedly presented statement as true compared to a statement that was presented for the first time. Notably, the length of the retention interval had a significant influence on the effect size: The truth effect was larger with a ten-minute than with a one-week interval after the first presentation. This finding is in contrast to results from Nadarevic and Erdfelder (2014) who report an increased effect size after a one-week compared to a ten-minute interval. However, in their study participants already had to provide truth judgments in the first exposure phase, whereas in the present study a semantic categorization task was used in this phase. Assumably, after a rather long interval previously made truth ratings of identical statements during the exposition phase are not easily available, and thus can lead to an increased truth effect in comparison to a relatively short retention interval, as in the study by Nadarevic and Erdfelder (2014). That a decreasing strength of memory can influence the truth effect was also shown in other studies, which implemented different retention interval lengths. For example, Garcia-Marques et al. (2015) found that participants rated contradictory statements to be more false compared to new statements after a relatively short interval of a few minutes, but judged those as more likely being true after a longer, one-week, interval. We assume that the time-related reduction of the truth effect in our data is also based on reduced memory strength after the long interval, which in turn, diminishes the relative processing fluency of repeated statements. The explanation remains speculative but seems well in line with (a) our analyses of response times, which show that the effect of repetition status was reduced after a longer retention interval, and (b) with Unkelbach and Rom’s (2017) assumption that a higher number of coherent mental activations, while processing a statement, is driving the fluency-based truth effect.

Based on Topolinski and Strack’s (e.g., 2009) theoretical considerations that a fluency experience triggers positive affect and that this affective state could be a primary cue used in decision-making processes, we further investigated whether fluency-related changes in affective states may be (co-)responsible for the truth effect. In our first experiment, after a retention interval of one week, the repetition-based truth effect was reduced for statements post-cued with a negative prime. This finding provides preliminary evidence that the impact of processing fluency on truth judgments cannot be considered as independent of the positive affect which is linked to this fluency experience. The results of our second experiment further support this assumption: Participants who received an alternative (pseudo-)source for experienced changes in affective states showed a reduced truth effect. Of course, the assumption that positive affect is a relevant component of the mechanism underlying the repetition-based truth effect does not imply that it is the only one. It needs to be further investigated to what extent positive affect constitutes the truth effect. Interestingly, fluency and fluency-triggered positive affect were also identified as determinants of judgments in other contexts (e.g., in respect of visual coherence, semantic coherence, and grammaticality; see Topolinski & Strack, 2009).

In Experiment 1, the influence of primes was only observed after the longer (one-week) retention interval. We speculate that in case of the shorter (ten-minute) interval the subtle affective priming may have exerted an insufficient influence in comparison to the fluency manipulation by repetition. Likewise, it is plausible that after the shorter retention interval subjects partially remembered presented statements from the exposure phase, whereas a conscious recognition seems less likely after the one-week interval within our experimental design. A significant interaction between negative valence and repetition status is especially to be expected when the effect of repetition status is mainly based on the perceived fluency (including its positive affective component) and not, or only marginally, on conscious memory.

As an alternative explanation for the findings of Experiment 1, one might assume that due to an assimilative vs. accommodative processing (process model by Bless & Fiedler, 2006) a positive affective state should promote and a negative affective state should reduce the reliance on the perceived processing fluency. However, we doubt that the applied short-term manipulations of the affective states, that were implemented after each statement presentation, are sufficient to decisively promote an assimilative vs. accommodative processing of the statements, and therefore influenced the truth judgments. As primes were presented after the statements, we rather assume that statements had already been well processed at the time of affect induction. Beyond that, according to the argumentation above, a positive affective state should promote the reliance on the perceived fluency, so that a stronger truth effect in the case of positive in comparison to neutral affective priming would be expected. However, no interaction effect between repetition status and positive valence was observed in Experiment 1.

In contrast to studies showing that a positive affective state alone can increase the likelihood of rating a statement as true (e.g., Garcia-Marques et al., 2004), we did not find any evidence for more “true” ratings for new statements in the case of positive compared to neutral priming in our first experiment. As we manipulated fluency and short-term affective states within participants in this study, it is possible that fluency was used as the primary cue for judging the truthfulness of the statements, which could have hampered the reliance on weaker cues such as a short-lasting positive affect induced by priming. This idea is further supported by the significant interactions of positive and negative affect with the preference for intuition after the one-week interval. Participants with a high preference for intuition, who typically include affective information in decision-making processes (Betsch, 2005), obviously even used the minimal affective information provided by the very briefly presented affective primes as cues for their truth judgments.

The individual results of our two experiments further demonstrate that the truth effect is moderated by the trait variables need for cognitive closure (Experiment 1 & 2) and preference for deliberation (Experiment 1). After the ten-minute retention interval, participants with a higher need for cognitive closure exhibited a stronger truth effect, and a higher preference for deliberation led to a reduction of the truth effect (Experiment 1). After a retention interval of one week, however, these interactions decreased. Results of Experiment 2 replicated the finding that individuals with a higher need for cognitive closure showed a stronger truth effect. Likewise, in line with our previous results, this effect was reduced after a longer retention interval (1 week). Different findings following various retention interval lengths may be, again, due to memory strength differences. It is plausible that, after a relatively short interval, memory traces are still strong, and thus the previous presentation is more likely to be recognized as the source of the familiarity feeling. This line of argumentation is supported by results of Arkes, Hackett, and Boehm (1989) demonstrating that the truth effect is enlarged when repeated statements are ascribed to sources outside of the experiment. That is, as long as the fluency can be attributed to the repeated presentation within the experiment, people with a higher preference for deliberation are probably less likely to base their truth judgments on perceived fluency, whereas people with a higher need for cognitive closure may think less about the “source” of the familiarity feeling and thus might rely more on it for their truth judgment. After one week, the source of the familiarity was probably less clear, which might have caused the interaction between repetition status and preference for deliberation, as well as between repetition status and need for cognitive closure, to decrease. When the feeling of familiarity could not be attributed to the source “experiment” anymore, a stronger reliance on perceived high fluency can be expected. In fact, fluency can be considered as a valid cue in the context of truth judgments and therefore, decision makers could be well advised to trust such cues when they have to decide under uncertainty (Reber & Unkelbach, 2010).

Our findings appear partially inconsistent relative to the recent results from De keersmaecker et al. (2020), who found no moderating effect of need for cognitive closure or preference for deliberation and intuition on the truth effect. For an adequate integration of the diverse findings, a closer look at the applied methods is crucial. As mentioned before, the authors did not find any evidence for effects of PI or PD on the size of the truth effect. At least with regard to PD we found a negative relationship in our first experiment, although the descriptive findings shown in Fig. 1 (for a clear illustration a median split for PD was accomplished) suggest that this effect is mainly based on different judgments for new statements. These conflicting results may be based on subtle methodological details (e.g., usage of the German version vs. the English version of the PID scale), or differences between samples (we used a German student sample and De keersmaecker et al., 2020, samples of Amazon Mechanical Turk workers). Indeed, across our two studies the role of PD in the context of the truth effect was also inconsistent. Thus, our results suggest PD as a candidate for an individual difference variable influencing the truth effect, but further research is needed to clarify its role. In our studies no significant influence of preference for intuition (PI) on the magnitude of the truth effect was observed. We, as well as De keersmaecker et al. (2020), used Betsch’s (2004) PI-Scale to assess which strategy people use when making decisions. Individuals with a high PI are assumed to be more likely to base their decisions on immediately available affective states. However, the typical materials for investigating the truth effect involve statements for which a high uncertainty about their truth should exist in the general population. Under these uncertain circumstances and in the absence of any other valid cue, both people with high and low preference for intuition probably rely on the fluency experience for their truth judgments.

Lastly, our findings in the context of need for cognitive closure and the truth effect, especially considering the different retention interval lengths, seem well in line with the data by De keersmaecker et al. (2020); the authors found no moderating role of need for cognitive closure regarding the truth effect after a (relatively long) retention interval spanning five to seven days (another interval length was not implemented in this respect). Beyond that, of course, some further methodological decisions may have also contributed to the varying results in this context, such as the use of different scales for measuring NCC; we used the German short scale by Schlink and Walther (2007) and De keersmaecker et al. (2020) the 15-item NCC scale by Roets and Van Hiel (2011). Like De keersmaecker et al. (2020), we informed the participants that true and false statements are presented during the exposure phase; different instructions in this respect (as in studies by Newman et al., 2020) can therefore be ruled out as a possible cause for the various findings.

For full transparency, we finally want to acknowledge that we collected data from young German adults, most of them university students. Therefore, the generalizability of the proposed effects to other age groups, other cultural contexts, or to groups with other educational backgrounds cannot be assessed from our data. Nonetheless, since the presence of the truth effect has been demonstrated in many studies from different labs with diverse kinds of samples, we believe that this is a widespread phenomenon, and we see no reasons to assume that the basic affective mechanisms and individual differences shown here should be limited to the specific groups examined in our studies.

## Conclusion

With the present research, we showed (1) that the repeated presentation of statements increased the probability that these statements were judged to be true (i.e., the truth effect), (2) that this truth effect was stronger, when the repetition interval was short (10 min) rather than long (1 week), (3) was reduced by negative post-cues (sad faces), as well as by the presence of an irrelevant source for changes in affective states, and (4) was increased by a higher need for cognitive closure.

The present research expands previous knowledge of the truth effect by bringing together manipulations of repetition status, retention intervals, affective post-primes (Experiment 1)/presence of an alternative source for changes in affective states (Experiment 2), and the assessment of dispositional variables.

## Figures and Tables

**Fig. E1:**
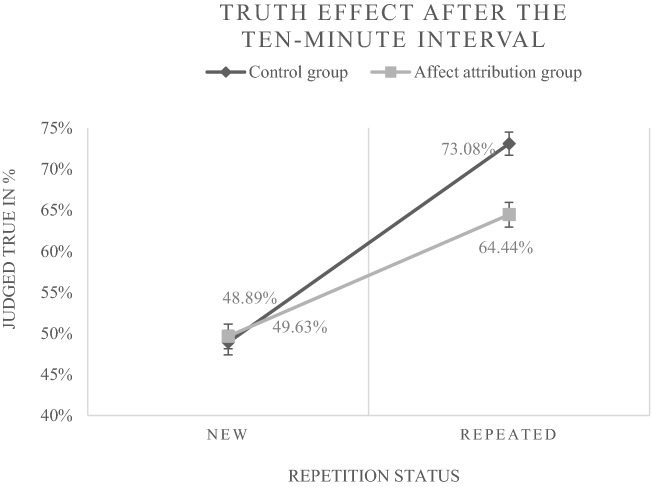
Percentages of "true" judgments for new (disfluent) and repeated (fluent) statements in the control and affect attribution group after the ten-minute interval (Experiment 2). Error bars represent standard errors

**Fig. E2:**
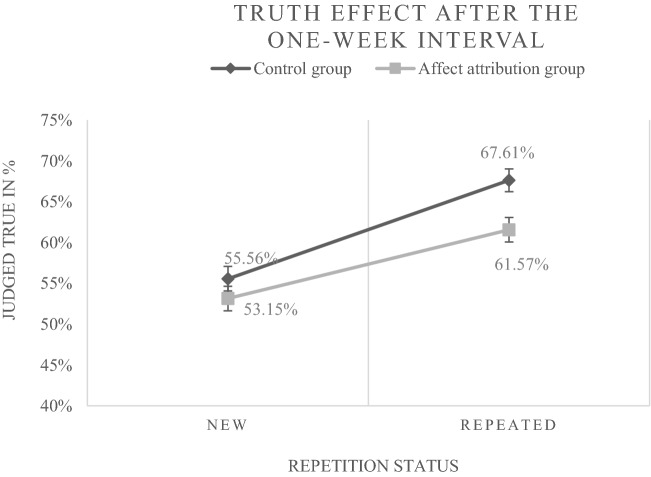
Percentages of "true" judgments for new (disfluent) and repeated (fluent) statements in the control and affect attribution group after the one-week interval (Experiment 2). Error bars represent standard errors

## Appendix A: additional analyses of confidence regarding truth judgments (Experiment 1)

For the analyses of confidence ratings, we used the lme4 R package (Bates et al., 2015) in combination with the lmerTest package (Kuznetsova, Brockhoff, & Christensen, 2017) for calculating *p*-values. A linear mixed model fit by maximum likelihood was used (*t*-tests use Satterthwaite approximations to degrees of freedom). As in the analyses concerning judgments of truth, models with random intercepts for participants and items were specified separately for the first and second judgment phase to investigate the questions of interest.

### Confidence ratings after the ten-minute interval

In Model 1c, including the level-1 predictors *repetition status*, *positive valence*, *negative valence* as well as all interactions involving these variables, only a main effect of repetition status was found (*b* = 0.681, *p* < 0.001). This finding indicates that repeated statements received higher confidence ratings than new statements. All results are displayed in Table A1.

**Table A1:** Multilevel modeling results for confidence ratings after the ten-minute interval (Experiment 1)

	Fixed Effects							
	Model 1c				Model 2c			
	*b*	*SE*	*t*	*p*	*b*	*SE*	*t*	*p*
Intercept	3.091	0.101	30.596	< 0.001***	3.029	0.093	32.535	< 0.001***
Repetition status	0.681	0.061	11.185	< 0.001***	0.743	0.035	21.259	< 0.001***
Valence (positive)	– 0.085	0.061	– 1.400	0.162	–	–	–	–
Valence (negative)	– 0.103	0.061	– 1.684	0.092	–	–	–	–
R.S. × V. (pos.)	0.120	0.086	1.386	0.166	–	–	–	–
R.S. × V. (neg.)	0.069	0.086	0.797	0.425	–	–	–	–
PI	–	–	–	–	0.134	0.165	0.814	0.418
PD	–	–	–	–	– 0.145	0.143	– 1.013	0.313
NCC	–	–	–	–	0.076	0.139	0.548	0.585
R.S. × PI	–	–	–	–	0.008	0.067	0.128	0.899
R.S. × PD	–	–	–	–	– 0.118	0.057	– 2.055	0.040*
R.S. × NCC	–	–	–	–	0.259	0.057	4.525	< 0.001***

*N* = 97

*R.S.*  repetition status, *V.*  Valence, *pos.*  positive, *neg.*  negative, *PI* preference for intuition, *PD* preference for deliberation, *NCC* need for cognitive closure

**p* < 0.05; ****p* < 0.001

Model 2c comprised the level-1 predictor *repetition status* and the level-2 predictors *preference for intuition*, *preference for deliberation* and *need for cognitive closure* as well as all cross-level interactions involving these variables. Results from this model are presented in Table A1. AIC and BIC decreases indicated an improved model fit for Model 2c compared to the Model 1c (Model 1c: AIC = 20370.1 & BIC = 20430.1; Model 2c: AIC = 20351.8 & BIC = 20425.1).

In addition to the main effect of *repetition status* (*b* = 0.743, *p* < 0.001), cross-level interactions of *repetition status* and *preference for deliberation* (*b* = – 0.118, *p* = 0.040) and of *repetition status* and *need for cognitive closure* (*b* = 0.259, *p* < 0.001) were observed. According to these results, the effect of *repetition status* on confidence ratings was weaker for people with a higher preference for deliberation but stronger for people with a higher need for cognitive closure.

### Confidence ratings after the one-week interval

When the level-1 predictors *repetition status*, *positive valence*, *negative valence* as well as all interactions involving these variables were included in Model 1d, only a main effect for *repetition status* was found (*b* = 0.225, *p* < 0.001). Again, repeated statements were rated with higher confidence than new statements. Results are displayed in Table A2.

**Table A2:** Multilevel modeling results for confidence ratings after the one-week interval (Experiment 1)

	Fixed Effects							
	Model 1d				Model 2d			
	*b*	*SE*	*t*	*p*	*b*	*SE*	*t*	*p*
Intercept	2.897	0.101	28.829	< 0.001***	2.900	0.093	31.052	< 0.001***
Repetition Status	0.225	0.057	3.949	< 0.001***	0.266	0.033	8.124	< 0.001***
Valence (positive)	0.004	0.057	0.065	0.948	–	–	–	–
Valence (negative)	0.005	0.057	0.088	0.930	–	–	–	–
R.S. × V. (pos.)	0.061	0.081	0.752	0.452	–	–	–	–
R.S. × V. (neg.)	0.062	0.081	0.765	0.444	–	–	–	–
PI	–	–	–	–	0.054	0.163	0.335	0.739
PD	–	–	–	–	– 0.142	0.141	– 1.007	0.316
NCC	–	–	–	–	0.236	0.136	1.730	0.087
R.S. × PI	–	–	–	–	0.019	0.063	0.305	0.761
R.S. × PD	–	–	–	–	– 0.007	0.055	– 0.119	0.906
R.S. × NCC	–	–	–	–	0.004	0.052	0.075	0.941

*N* = 97

*R.S.*  repetition status, *V.*  Valence, *pos.*  positive, *neg.*  negative, *PI* preference for intuition, *PD* preference for deliberation, *NCC* need for cognitive closure

****p* < 0.001

In Model 2d *repetition status* was included as the only level-1 predictor alongside the level-2 predictors *preference for intuition*, *preference for deliberation* and *need for cognitive closure* as well as all cross-level interactions involving these variables. Again, only a main effect of *repetition status* was observed (*b* = 0.266, *p* < 0.001; see Table A2 for all results). AIC and BIC increases indicated a deteriorated model fit for the second model compared to the first model (Model 1d: AIC = 19660.2 & BIC = 19720.2; Model 2d: AIC = 19662.0 & BIC = 19735.4).

## Appendix B: Additional analyses of confidence regarding truth judgments (Experiment 2)

For the analyses of confidence ratings, we used the lme4 R package (Bates et al., 2015) in combination with the lmerTest package (Kuznetsova et al., 2017) for calculating *p*-values. A linear mixed model fit by maximum likelihood was used (*t*-tests use Satterthwaite approximations to degrees of freedom). As in the analyses concerning judgments of truth, models with random intercepts for participants and items were specified.

Model 1b comprised the predictors *repetition status*, *affect attribution* and *judgment phase* as well as all interactions involving these variables. Results show a main effect of repetition status (*b* = 0.541, *p* < 0.001) as well as an interaction between repetition status and judgment phase (*b* = – 0.520, *p* < 0.001). This finding indicates that repeated statements received higher confidence ratings than new statements, and that a longer time interval between the first exposure and the later judgment phases reduced the size of this effect. All results are displayed in Table B1.

**Table B1:** Multilevel modeling results for confidence ratings (Experiment 2)

	Fixed effects							
	Model 1b				Model 2b			
	*b*	*SE*	*t*	*p*	*b*	*SE*	*t*	*p*
Intercept	2.757	0.121	22.818	< 0.001***	2.794	0.093	29.897	< 0.001***
Repetition Status	0.541	0.041	13.238	< 0.001***	0.497	0.030	16.332	< 0.001***
Affect Attribution	0.076	0.162	0.473	0.638	–	–	–	–
Judgment Phase	– 0.098	0.058	– 1.693	0.091	– 0.178	0.043	– 4.131	< 0.001***
R.S. × A.A	– 0.009	0.059	– 0.158	0.875	–	–	–	–
R.S. × J.P.	– 0.520	0.082	– 6.359	< 0.001***	– 0.427	0.061	– 7.015	< 0.001***
A.A. × J.P.	– 0.125	0.083	– 1.505	0.133	–	–	–	–
R.S. × A.A. × J.P.	0.125	0.118	1.065	0.287	–	–	–	–
PI	–	–	–	–	0.111	0.134	0.832	0.408
PD	–	–	–	–	0.101	0.144	0.702	0.485
NCC	–	–	–	–	– 0.156	0.146	– 1.065	0.290
Repetition Status × PI	–	–	–	–	0.138	0.050	2.758	0.006**
Repetition Status × PD	–	–	–	–	0.112	0.054	2.096	0.036*
Repetition Status × NCC	–	–	–	–	0.308	0.055	5.606	< 0.001***
R.S. × PI × J. P.	–	–	–	–	– 0.212	0.071	– 2.982	0.003**
R.S. × PD × J. P	–	–	–	–	0.073	0.076	0.953	0.341
R.S. x NCC x J. P.	–	–	–	–	0.095	0.078	1.217	0..224

*N* = 75 (Model 1b); *N* = 69 (Model 2b)

*R.S.*  repetition status, *J.P.* judgment phase, *A.A.* affect attribution, *PI* preference for intuition, *PD* preference for deliberation, *NCC* need for cognitive closure

**p* < 0.05; ***p* < 0.01; ****p* < 0.001

In Model 2b, in addition to the predictors *repetition status*, *judgment phase*, and *repetition status × judgment phase*, the variables *preference for intuition*, *preference for deliberation* and *need for cognitive closure*, cross-level interactions involving these and repetition status as well as three-way interactions involving these dispositional variables, repetition status and judgment phase were added. Results show a main effect of repetition status (*b* = 0.497, *p* < 0.001), of judgment phase (*b* = – 0.178, *p* < 0.001), as well as an interaction between repetition status and judgment phase (*b* = – 0.427, *p* < 0.001). In addition, the cross-level interaction of repetition status and need for cognitive closure (*b* = 0.308, *p* < 0.001), and of repetition status and preference for intuition (*b* = 0.138, *p* = 0.006) was found. A significant three-way interaction between repetition status, preference for intuition, and judgment phase (*b* = – 0.212, *p* = 0.003) indicated that the interaction effect of repetition status and preference for intuition was influenced by the length of the retention interval. Furthermore, an interaction of repetition status and preference for deliberation was found, although the direction of the effect was not as expected (*b* = 0.112, *p* = 0.036). Table B1 shows all results.

Decreases of AIC and BIC indicate an improved model fit for the second model compared to the first model (Model 1b^16^: AIC = 29439.1 & BIC = 29516.3; Model 2b: AIC = 29394.3 & BIC = 29506.7).

## Appendix C: discussion—confidence regarding truth judgments

Finally, we tested the influences of all predictors discussed in the context of truth judgments on the confidence about truth judgments. Although it is known that fluency can influence confidence (e.g., Alter et al., 2007), Koch and Forgas (2012) reported that processing fluency did not simultaneously influence both truth judgments and confidence about those judgments. In contrast, we found that repetition (a source of perceptual and conceptual fluency) influenced truth judgments and confidence about these ratings in both experiments. As in the context of truth judgments, this effect was reduced by a longer time interval. The differences to Koch and Forgas’ (2012) results could be due to differences in fluency manipulations, as in their experiment a purely perceptual processing fluency manipulation was used. According to Parks and Toth (2006), conceptual processing fluency has a much larger impact on truth judgments than perceptual processing fluency. Thus, it is plausible that the influence of conceptual fluency is also larger on confidence about those truth judgments.

Moreover, we found that the effect of repetition on confidence (as on the truth ratings themselves) was stronger for people with a higher need for cognitive closure in both experiments. As mentioned above, we assumed that people with a high NCC, who prefer unambiguous situations and make quick decisions with a high subjective confidence (Schlink & Walther, 2007), should be more likely to base their truth judgments on instantly perceived fluency. In line with this, it is reasonable to assume that people with a high NCC also tend to be more confident in their judgments which they based on instantly perceived processing fluency. For individuals with a higher preference for deliberation, we found inconsistent results: in Experiment 1 a higher preference for deliberation was associated with a weaker and in Experiment 2 with a stronger effect of repetition status on confidence ratings. Inconsistent results were also observed regarding preference for intuition: whereas in our second experiment a moderating role of PI on the effect could be found, this was not the case in our first experiment. Thus, our results suggest PD and PI as potential candidates for an individual difference variable influencing the effect of repetition on confidence, but further research is needed to clarify their role.

However, no main effects were found for positive valence or negative valence on confidence ratings. Likewise, no significant interaction between repetition status and positive valence or between repetition status and negative valence was revealed in this context (Experiment 1). Consistent with this finding, the provision of an alternative source for changes in affective states had no impact on the effect of repetition status as well. Based on these results, we conclude that fluency-related changes in affective states are (co-)responsible for the truth effect, but that confidence judgments may not be simultaneously influenced by an affective component of the fluency experience.

## Appendix D: additional analyses of potentially confounding factors

Since the photos of the KDEF database seem to be a little more expressive compared to the photos of the databases RaFD and WSEFEP, possible effects of the photo sets used were examined separately for both judgment phases. For this purpose, the level-1 predictors repetition status, positive valence, negative valence and the dichotomous variable *photo set* (coded –0.5 for the first photo set which included KDEF images and + 0.5 for the second photo set which included RaFD and WSEFEP images) as well as all interactions involving these variables were integrated into the model to predict the log-odds of judging a statement as true. With regard to both judgment phases no significant main or interaction effects concerning the variable *photo set* were found.

According to a post-experimental inquiry, twelve participants indicated that they had seen changing emotions in the portraits but had no appropriate hypothesis regarding the aim of the study. Nineteen persons made the appropriate assumption that the study aim was to examine the influence of repetition on truth judgments and 28 persons made the appropriate assumption that one purpose of the study was to examine the influence of emotional facial expressions on their judgments; nine of these persons assumed that both influencing factors (influence of repetition and emotional facial expressions) were examined. Some of the participants who assumed that the study was used to examine the influence of emotional facial expressions on judgments, did not describe the emotional facial expressions when asked for optical abnormalities in an additional question. However, based on their hypotheses regarding the aim of this study, it can be deduced that they also noticed the affective priming.

It was examined whether the awareness of the affective priming had any influence on the present results. In total, 57 persons did not notice the affective priming, while 40 persons did notice the affective priming. The level-1 predictors repetition status, positive valence, negative valence and the dichotomous variable *awareness of the affective priming* (coded – 0.5 for persons who did not notice the affective priming and + 0.5 for persons who noticed the affective priming) as well as all interactions involving these variables were integrated into the model to predict the log-odds of judging a statement as true. With regard to both judgment phases no significant main or interaction effects concerning the variable *awareness of the affective priming* were found.

In addition, it was examined whether the existence of appropriate hypotheses regarding the aim of this study had any influence on the fundamental results. In total, 38 persons had at least one appropriate hypothesis regarding the study aim. The level-1 predictors repetition status, positive valence, negative valence, the dichotomous variable *existence of an appropriate hypothesis regarding the aim of this study* (coded − 0.5 for persons who did not describe any appropriate hypothesis and + 0.5 for persons who stated at least one appropriate hypothesis regarding the aim of this study) as well as all interactions involving these variables were integrated into the model to predict the log-odds of rating a statement as true. With respect to the ten-minute retention interval, concerning the variable *existence of an appropriate hypothesis regarding the aim of this study* no interaction effects were found but there was a main effect (*b* = – 0.395, *p* = 0.017). When excluding those persons who had at least one correct hypothesis regarding the aim of this study and entering the level-1 predictors repetition status, positive valence, negative valence as well as all interactions involving these variables, the pattern of results did not change compared to the results of an analysis with all 97 participants. With respect to the one-week interval, no significant main or interaction effects concerning the variable *existence of an appropriate hypothesis regarding the aim of this study* were found.

Furthermore, five persons declared that they have informed themselves about the truth content of statements between the first and the second test session. All statements in question were part of the first judgment phase and subsequently were not repeated in the second judgment phase, which is why this piece of information is irrelevant for the analyses.

## Appendix E: Separate analyses for the first and second judgment phase (Experiment 2)

See Table E1, Figure E1, Table E2, Figure E2.

**Table E1:** Multilevel logistic modeling results for the prediction of "true" responses after the ten-minute interval (Experiment 2)

	Fixed Effects							
	Model 1c				Model 2c			
	*b*	*SE*	*z*	*p*	*b*	*SE*	*z*	*p*
Intercept	– 0.044	0.124	– 0.350	0.726	– 0.085	0.127	– 0.669	0.503
Repetition Status	1.183	0.096	12.341	< 0.001***	1.194	0.099	12.027	< 0.001***
Affect Attribution	0.028	0.162	0.174	0.862	0.102	0.167	0.610	0.542
R.S. × A.A.	– 0.488	0.133	– 3.666	< 0.001***	– 0.567	0.139	– 4.082	< 0.001***
PI	–	–	–	–	0.119	0.137	0.864	0.388
PD	–	–	–	–	– 0.027	0.147	– 0.183	0.855
NCC	–	–	–	–	0.003	0.150	0.020	0.984
R.S. × PI	–	–	–	–	0.138	0.116	1.192	0.233
R.S. × PD	–	–	–	–	0.041	0.122	0.331	0.740
R.S. × NCC	–	–	–	–	0.341	0.131	2.603	0.009**

*N* = 75 (Model 1c); *N* = 69 (Model 2c)

*R.S.*  repetition status, *A.A.* affect attribution, *PI* preference for intuition, *PD* preference for deliberation, *NCC* need for cognitive closure

***p* < 0.01; ****p* < 0.001

**Table E2:** Multilevel logistic modeling results for the prediction of "true" responses after the one-week interval (Experiment 2)

	Fixed Effects							
	Model 1d				Model 2d			
	*b*	*SE*	*z*	*p*	*b*	*SE*	*z*	*p*
Intercept	0.267	0.116	2.311	0.021*	0.278	0.122	2.285	0.022*
Repetition Status	0.578	0.092	6.319	< 0.001***	0.561	0.096	5.863	< 0.001***
Affect Attribution	– 0.124	0.147	– 0.844	0.399	– 0.114	0.155	– 0.730	0.465
R.S. × A.A.	– 0.194	0.130	– 1.491	0.136	– 0.196	0.136	– 1.437	0.151
PI	–	–	–	–	0.049	0.129	0.378	0.706
PD	–	–	–	–	– 0.136	0.137	– 0.988	0.323
NCC	–	–	–	–	0.124	0.140	0.890	0.374
R.S. × PI	–	–	–	–	0.106	0.115	0.917	0.359
R.S. × PD	–	–	–	–	0.188	0.122	1.549	0.121
R.S. × NCC	–	–	–	–	0.061	0.123	0.496	0.620

*N* = 75 (Model 1d); *N* = 69 (Model 2d)

*R.S.*  repetition status, *A.A.* affect attribution, *PI* preference for intuition, *PD* preference for deliberation, *NCC* need for cognitive closure

**p* < 0.05; ****p* < 0.001

## Footnotes

1 All additional analyses in the context of confidence about truth judgments and the subsequent discussion of these results can be found in the appendix (see Appendix A–C).

2 In multilevel analysis, level-2 sample size is often most important for power (Maas & Hox, 2005). Simulation studies by Maas and Hox (2005) indicate that in multilevel modeling, parameter estimates are of sufficient accuracy with a minimum of 50 level-2 units (see, in addition, Paccagnella, 2011). The same value of a minimum of 50 level-2 units is advised for multilevel logistic regression models (Moineddin, Matheson, & Glazier, 2007) to achieve valid estimates. With 97 level-2 units (participants) and 60 units per cluster (observations per judgment phase) the present study was well in line with all of these requirements.

3 This presentation time was shown to be appropriate in the conducted pretests to ensure the complete reading and comprehension of the selected statements.

4 Persons probably base their judgments increasingly on the experienced processing fluency when they are under time pressure. This is important to consider as one of our main goals was to investigate whether fluency-related changes in affective states may be (co-)responsible for the truth effect. Nevertheless, we assume that this form of instruction, that stresses accuracy and speed simultaneously, does not substantially influence the relationship between PID and the truth effect, which we were also interested in.

5 Outliers in the reaction time distribution—defined as lying more than 1.5 interquartile ranges below the first or above the third quartile of the logarithmized reaction time distribution—were removed prior to this analysis.

6 The optimizer *bobyqa* (Powell, 2009) was applied for this analysis.

7 Numbers (1 & 2) indicate models with level-1 predictors or level-1 and level-2 predictors, respectively, the letters (a, b, c, & d) serve to differentiate between the models built for different judgment phases and/or with different variables included (analyses of confidence ratings can be found in Appendix A).

8 The optimizer *bobyqa* (Powell, 2009) was applied for this analysis.

9 In multilevel analysis, level-2 sample size is often most important for power (Maas & Hox, 2005). Simulation studies by Maas and Hox (2005) indicate that in multilevel modeling, parameter estimates are of sufficient accuracy with a minimum of 50 level-2 units (see, in addition, Paccagnella, 2011). The same value of a minimum of 50 level-2 units is advised for multilevel logistic regression models (Moineddin et al., 2007) to achieve valid estimates. With 75 level-2 units (participants) and 60 units per cluster (observations per judgment phase) the present study was well in line with all of these requirements.

10 For this analysis, as well as for all following analyses including the variables NCC, PD and PI, statistical outliers were identified using the 1.5 × IQR rule; subsequently the data of six participants was excluded.

11 Outliers in the reaction time distribution—defined as lying more than 1.5 interquartile ranges below the first or above the third quartile of the logarithmized reaction time distribution—were removed prior to this analysis.

12 Numbers (1 & 2) indicate models with different predictors (dispositional variables included/not included), the letters (a & b) serve to differentiate between the models built for truth judgments and confidence ratings (analyses of confidence ratings can be found in Appendix B).

13 For full transparency, we added separate analyses for the two judgment phases in Appendix E. Figures E1 and E2 illustrate the frequencies underlying the interaction between repetition status and affect attribution after the ten-minute and one-week retention interval, respectively.

14 The interaction (repetition status × NCC) was, like the truth effect in general, reduced after the longer time interval (1 week). This finding is well in line with the data pattern of Experiment 1.

15 For this comparison, Model 1a was tested with the same sample size as Model 2a (*N* = 69; the present pattern of results did not change compared to the analysis with all 75 participants).
